# From Signal to Image: Enabling Fine-Grained Gesture Recognition with Commercial Wi-Fi Devices

**DOI:** 10.3390/s18093142

**Published:** 2018-09-18

**Authors:** Qizhen Zhou, Jianchun Xing, Wei Chen, Xuewei Zhang, Qiliang Yang

**Affiliations:** National Defense Engineering College, Army Engineering University of PLA, Nanjing 210007, China; zhouqizhen2016@163.com (Q.Z.); xjc@893.com.cn (J.X.); johnny185@126.com (W.C.); zxwlucky@163.com (X.Z.)

**Keywords:** gesture recognition, channel state information, image processing, deep learning

## Abstract

Gesture recognition acts as a key enabler for user-friendly human-computer interfaces (HCI). To bridge the human-computer barrier, numerous efforts have been devoted to designing accurate fine-grained gesture recognition systems. Recent advances in wireless sensing hold promise for a ubiquitous, non-invasive and low-cost system with existing Wi-Fi infrastructures. In this paper, we propose *DeepNum*, which enables fine-grained finger gesture recognition with only a pair of commercial Wi-Fi devices. The key insight of *DeepNum* is to incorporate the quintessence of deep learning-based image processing so as to better depict the influence induced by subtle finger movements. In particular, we make multiple efforts to transfer sensitive Channel State Information (CSI) into depth radio images, including antenna selection, gesture segmentation and image construction, followed by noisy image purification using high-dimensional relations. To fulfill the restrictive size requirements of deep learning model, we propose a novel region-selection method to constrain the image size and select qualified regions with dominant color and texture features. Finally, a 7-layer Convolutional Neural Network (CNN) and SoftMax function are adopted to achieve automatic feature extraction and accurate gesture classification. Experimental results demonstrate the excellent performance of *DeepNum*, which recognizes 10 finger gestures with overall accuracy of 98% in three typical indoor scenarios.

## 1. Introducti on

The interactions with smart sensors have advanced to an unprecedented extent in this day and age [1]. Body gesture, as a novel communication modality, is widely adopted in HCI for its natural and straightforward properties [2]. To realize practical gesture recognition, tremendous efforts have been made both in terms of accuracy and granularity. Most schemes achieve the goals based on dedicated motion sensors [3] or pre-installed depth cameras (e.g., RGB-D sensor [4], Kinect [5] and Leap motion [6]). Despite their high accuracy in tracking subtle finger motions, they have intrinsic limitations such as whole-day intrusive equipment, light and Line-of-Sight (LoS) condition, potential privacy issues and extra installation cost. We look forward to a ubiquitous, non-invasive, low-cost finger gesture recognition system, which could bring real advantages to a user-friendly HCI. For instance, if a housewife enjoys a bubble bath, it is preferable for her to lie in the bathtub and control intelligent appliances remotely (e.g., aquastat, light and audio clusters), rather than worry about the waterproofness of precise instruments or privacy leakage caused by cameras.

Recent advances in Wi-Fi-based in-air gesture recognition shed light on the potential of realizing fine-grained gesture recognition with almost-everywhere commercial Wi-Fi devices. Generally, these systems reuse pervasive Wi-Fi signals by capturing and analyzing informative signal characteristics induced by human motions. Early attempts were devoted to deriving motion-induced Doppler shifts [7], temporal amplitude profiles [8] or angle-of-arrival (AOA) values [8] to detect minute gestures, yet these prototypes were only implemented on specialized hardware (SH). Pioneer works extract either coarse-grained Received Signal Strength (RSS) [10] or fine-grained Channel State Information (CSI) [11,12,13,14,15] from off-the-shelf Wi-Fi devices and recognize micro-movements using temporal profiles. Nevertheless, due to the imperfect commercial NICs, it is challenging to derive invariant motion-induced signatures from superimposed signals (i.e., reflected off both environment dynamics and human gestures) with limited bandwidth and antennas. Moreover, traditional classification methods like Dynamic Time Warping (DTW) and k-Nearest Neighbor (k-NN) cannot handle the increasing number of gesture categories, and yield lower accuracy and computation efficiency [16]. A deep learning-based method is a promising alternative, since it achieves automatic feature extraction and precise gesture classification. Recent literature on wireless localization [17,18] and activity recognition [19,20] has demonstrated the viability of wireless gesture recognition using Deep Neural Networks (DNN). The key insight is that multiple hidden layers and neuron units could extract essential features from seemingly alike CSI measurements. However, an exponential number of full-connected elements may require burdensome training efforts [21].

In this paper, we propose a fine-grained finger gesture recognition system utilizing a deep CNN architecture, named *DeepNum*. Compared with our previous DNN-based version [21], *DeepNum* employs a more powerful architecture by extracting prominent features using multiple convolutional filters and reducing the training cost via parameter sharing, and thus possesses better performance. More importantly, we incorporate the ideology of image processing and design a novel pipeline to transfer raw CSI measurements into qualified radio images. As far as we know, *SignFi* [23] is the most closely related work in terms of leveraging CNN to recognize hand and finger movements. However, we argue that *DeepNum* still outperforms *SignFi* in the following two aspects.

- First, we are the first to embed CSI segmentation in a CNN-based system. To achieve real-time gesture recognition, detecting transition points of human movements and segmenting CSI frame into slices are critical processes. Yet it is non-trivial to import varied-size segments into the CNN model directly, since the input layer requires fixed-size input. *SignFi* simplifies this problem and only collects 200 packets for each gesture, which cannot be practical in real scenarios. To address this problem, we present a novel method to limit the width and height of regions of interest, and design a region-selection indicator to evaluate the significance of regions.
- Secondly, we construct depth radio images with sensitive CSI information. In *SignFi*, CSI measurements of both amplitude and phase are preprocessed and fed into CNN model. In contrast, we construct depth radio images with only amplitude from the most sensitive antenna and conjugate-multiplied phase from antenna pairs. In addition, spatial relations are fully considered to eliminate image noises. Therefore, we were able to preserve the most discriminative features of finger gestures while discarding polluted and insensitive signatures.

We implement *DeepNum* on commercial Wi-Fi infrastructures equipped with slightly modified NICs and evaluate the system performance in three typical indoor environments. Extensive experiments are conducted to show that *DeepNum* could achieve overall accuracy of 98%, slightly exceeding *SignFi* and obviously better than *WiFinger* in predefined scenarios. A variety of experimental studies are performed to test the influencing factors and system robustness. In the future, we envision *DeepNum* will promote the integration of wireless sensing applications with cutting-edge deep learning techniques.

Our contributions are summarized as follows.

- **From macro-movements to micro finger gestures.***DeepNum* enables fine-grained finger gesture recognition using only a pair of commercial Wi-Fi devices. Extensive experiments are conducted to validate the system performance and show superior performance compared with the state of the art.
- **From statistics to deep learning.** We propose a CNN-based deep learning framework to achieve automatic feature extraction and robust gesture classification without labor-intensive training efforts. Our solution breaks the constraints of the size requirement in the existing CNN model.
- **From CSI signals to deep radio images.** We present a set of novel methods to transfer signal processing into image processing, including raw CSI pre-processing, high-dimensional radio image construction, and image purification using spatial relations.

The subsequent sections are organized as follows. We firstly review the related work in Section 2, and then introduce preliminaries in Section 3. In Section 4, we present the system overview, followed by the detailed design of the signal processing and image processing in Section 5 and Section 6, respectively. Experimental results and evaluations are reported in Section 7. Finally, we conclude our work in Section 8.

## 2. Related Work

In this section, we introduce the source of our inspiration and state-of-the-art related works by dividing literatures into two parts: *Wi-Fi-based Gesture Recognition* and *Deep learning-based Wi-Fi Sensing* applications.

### 2.1. Wi-Fi-Based Gesture Recognition

As an early attempt, *WiSee* [7] shows the potential of discerning hand gestures by leveraging micro-level Doppler shifts using communication-based wireless signals from USRPs. *AllSee* [8] extracts gesture information from ambient radio frequency signals, like RFID and TV transmissions, to achieve low-complexity hand recognition. *RF-IDraw* [8] reveals the capability of high-resolution gesture tracking with a limited number of RFID antennas. NVIDIA [24] overcomes the light-sensitivity in vision-based gesture recognition by combining a FMCW radar. Google [25] implements an end-to-end fine gesture interaction system on millimeter-wave radar. All of the above systems exhibit promising prospects; however, specialized hardware (SH) is not pervasive in our daily life, so the promotion of SH-based systems may generate extra installation cost.

Recent decades have witnessed the prevalence of Wi-Fi devices and Wi-Fi-based sensing technologies. *WiGest* [10] leverages the RSS variations from unmodified Wi-Fi devices to indicate hand motions. Since CSI is tractable on slightly modified Wi-Fi NICs (like Intel 5300 [26] and Atheros 9390 [27]), users could easily acquire it from existing Wi-Fi infrastructures, avoiding extra deployment effort and installation cost. With subcarrier-level information, both accuracy and granularity of Wi-Fi-based sensing applications are improved. Qian et al. proposes *Widar* [28] and *Widar 2.0* [29] to track human speed and walking path. Wang et al. utilizes phase difference to detect the state transition in the case of falling [30]. Wang et al. exploits the distinctive spectrogram of human walking to discern different gait patterns [31]. Guo et al. developed a smart human dynamics monitoring system to estimate participant number, human density and walking speed [32]. The aforementioned works mainly focus on whole-body macro-movements and design hand-crafted features for specific activities. However, finger gesture recognition can be more challenging, since micro-movements imprint fewer characteristics in the received signals.

*WiDraw* [11] is inspired by the shadow effect caused by hand movements and then estimates AOA for trajectory tracking, yet it requires dense deployment of Wi-Fi devices. *WiKey* [12] illuminates a follow-up to recognizing subtle motions using temporal CSI waveforms. *WiFinger* [13,14] resorts to Discrete Wavelet Transform (DWT) for multi-layer analysis and DTW for efficient profile mapping. *WiAG* [15] makes further contributions in the position and orientation recognition of hand movements with a translation function. All of these state-of-the-art technologies achieve considerable results in specific scenarios, but they circumvent the exploitation of phase information, which may decrease the sensitivity and recognition accuracy. Additionally, classification algorithms like k-NN or DTW cannot fulfill the demands of an increasing number of gesture data [16]. Different from the above work, *DeepNum* leverages both sensitive amplitude and phase information, and then builds a deep learning-based framework dealing with growing gesture instances.

### 2.2. Deep Learning-Based Wi-Fi Sensing

We conduct surveys on deep learning-based applications and then introduce some successful examples with Wi-Fi. Deep learning techniques have been proposed since the early 21st century [33], and have earned great repute for their marvelous performance in image manipulation [34], natural language recognition [35] and mobile data processing [36]. In particular, we expect an up-and-coming trend when Wi-Fi encounters deep learning. The key insight is that deep learning techniques possess the capabilities of exploring the discriminative patterns from seemingly alike signal measurements.

*DeepFi* [17] takes the first step by applying DNN in wireless localization, then trains the optimal weights from CSI magnitude and restores them as fingerprints. Zhang et al. [18] updates the model by introducing a Hidden Malkov Model (HMM)-based fine tuner to smooth RSS-based localizations. Shi et al. [19] proposes a DNN-based user authentication scheme with preprocessed time and frequency features. Wang et al. [20] transforms the CSI matrix into 2-dimensional radio images and constructs image-based feature maps for the input of DNN. Due to the training complexity, we turn to CNN, since it only extracts features from local fields and reduces training cost via global parameter sharing. *CiFi* [37] collects equal-length CSI data and inputs AOA-based images to CNN. *WiQ* [38] applies CNN to train elaborate-selected feature maps since driver gestures generate distinguishable features in ambient USRP signals. *SignFi* is the most closely related paper to our work, and adopts a 9-layer CNN to complete 276 sign gesture recognition. However, to fulfill the requirement of deep learning architecture, all the above deep learning-based works simplify the problem by constructing matrices (tensor) with fixed length. For example, *SignFi* collects 200 samples of 3 × 30 subcarriers for each sign gesture, so that it can construct a 3-dimensional tensor with the fixed size of (3, 30, 200). However, it is not practical, since we do not know where the gesture begins and how long the duration is in wireless sensing. We seek to find the solutions in the visual domain, yet they only target a small span (e.g., 224 to 180 [39]), while the size of the CSI segments could vary significantly. In contrast to all these schemes above, *DeepNum* does not require fixed-length records or hand-crafted segmentations, which is more proper for practical use. Moreover, we design a set of novel image-processing methods, including action segmentation, radio image construction, and deep image sanitation, which all contribute to the capabilities of CNN-based classification.

## 3. Preliminaries

*DeepNum* was devised to achieve contactless finger gesture recognition with only a pair of commercial Wi-Fi devices. To build the relationship between *micro finger movements* and *slight signal distortions*, prior to this, we must understand the background knowledge of CSI, make motivation clear and pinpoint concomitant challenges.

### 3.1. Channel State Information

The dense deployment of Wi-Fi devices enables low-cost, easily deployed and fine-grained gesture recognition. The reason lies in the following three aspects: (1) we can reuse ubiquitous Wi-Fi signals with existing Wi-Fi networks; (2) we can easily obtain physical layer channel frequency response (CFR) of multiple subcarriers in the format of CSI, extracted from slightly modified Wi-Fi NICs; (3) modern Wi-Fi devices support Multiple-Input Multiple-Output (MIMO), which provides abundant spatial information from multiple trans-receiver pairs. Therefore, we are able to obtain subcarrier-level movement information from raw CSI measurements. Letting *y* and *x* denote the received signal and transmitting signal, the relation between *y* and *x* can be modeled as:

(1) y=Hx+noise,

where *H* represents the complex value of CFR, depicting the properties in the subcarrier level. Supposing that we acquire CSI measurement *H_t_* reported by Intel 5300 NIC for 1 × 3 frames (i.e., 1 and 3 are the numbers of transmitting and receiving antennas, respectively) at time *t*, then we can obtain CSI values for a total of 90 subcarriers, as:

(2) $$H_{t}={\left[H_{t}^{1},\ldots ,H_{t}^{n},\ldots ,H_{t}^{90}\right]}^{T},1\leq n\leq 90,$$

since CFR for a wireless channel also quantifies the variance of amplitude and phase response for a subcarrier, *H_t_* of the *n*-th subcarrier can be defined as:

(3) $$H_{t}^{n}=A_{t}^{n}e^{j\theta_{t}^{n}},$$

where *A_t_^n^* and *θ_t_^n^* represents the amplitude and phase of the *n*-th subcarrier at time *t*.

### 3.2. Motivation and Challenges

**Motivation**. Noting that a continuous CSI data matrix could be arranged as a set of amplitude and phase values, it is a natural choice to redesign multi-dimensional CSI tensors and explore deep relations across space. Zheng et al. [40] and Wang et al. [20] build 2-dimensional radio images using magnitude strength with row (time) as the x-axis and column (channel) as the y-axis. *SignFi* [23] converts raw CSIs into a 3-dimensional tensor, taking all amplitude and phase information into account. We try to take one step forward by constructing discriminative 3-dimensional CSI radio images and applying a set of image-processing methods. The motivation is strong and clear, and we summarize it in three layers. Firstly, 3-dimensional images (color images) naturally provide more informative features compared with 2-dimensional matrices (gray images), which contributes to discerning slight finger movements. Secondly, 3-dimensional image processing can deal with multiple channels and subcarriers simultaneously. If we apply SVD or Principal Component Analysis (PCA) [12] to each matrix separately, the channel correlation may be lost. Thirdly, the CNN model is ideal for extracting invariant features within high-dimensional data (across space), yet traditional DNN induces higher computation costs and lower accuracy. Therefore, we are inspired to bring image-based ideas into traditional signal-based wireless sensing and drive the progress of existing gesture recognition systems.

**Challenges**. The basic idea of this paper is to characterize slight signatures inherited from finger movements through image-based deep learning methods. To translate these high-level motivations into a practical system, we encounter the following challenges and summarize them with a few keywords.

1. *Raw material matters*. How to construct a discriminative 3-dimensional radio image with representative motion information?
2. *Relation appeals*. How to manipulate noisy radio images efficiently by leveraging the multi-channel correlations?
3. *CNN model requirements*. How to resolve the contradiction between fixed-size model requirement and variable-size image construction?

## 4. System Overview 

**Figure 1:**
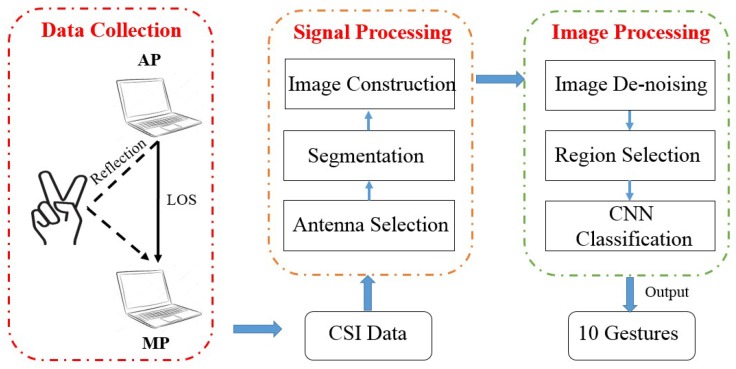
The system architecture of *DeepNum*.

To answer the above questions, we illustrate a processing procedure as is shown in Figure 1, which can be divided into three parts: *Data Collection*, *Signal Processing* and *Image Processing*. In the *Data Collection* step, we use a pair of laptops equipped with wireless cards to collect raw CSI measurements. One laptop, acting as the AP, transmits CSI packets with only one antenna. Another laptop, acting as the MP, receives CSI continuously with three antennas.

For the *Signal Processing* part, we first select a sensitive antenna as a reference and utilize the wavelet energy of only amplitude information to segment the continuous CSI sequence. Notice that we do not adopt the traditional signal de-noising methods here for the sake of simplicity and information integrity. Then, we construct a 3-dimensional tensor (called a 3-channel radio image) with two relative phase images and one amplitude image (for challenge 1).

For the *Image Processing* part, we make our contributions by designing novel processing methods for radio images. First of all, considering the informative features involved in channel correlations, we sanitize noisy radio images by leveraging Higher-Order Singular Value Decomposition (HOSVD) for high-dimensional principal components (for challenge 2). Then we borrow the idea of selective search in the image domain, extracting fixed-size image regions from varied-size radio images based on discriminative color and texture features with weighted values. Extracted image regions could fit the bill of the input layer in a deep learning architecture (for challenge 3). Finally, a 7-layer CNN and SoftMax function are applied to output classified labels of finger gestures.

## 5. Signal Processing 

Raw CSI measurements provided by commercial Wi-Fi devices cannot be utilized directly for micro-movement recognition. This is because motion characteristics involved in signal distortions can be sheltered by irrelevant interferences. Therefore, we only utilize the amplitude from the most sensitive antenna and relative phase information from conjugated antenna pairs. Additionally, as APs continuously broadcast CSI signals into the air, it is necessary to detect and segment motion-induced CSI frames in a time that is computationally efficient, so we leverage robust wavelet energy to indicate state transitions. In addition, we argue that manipulating CSI amplitude and phase information jointly preserves more available signatures; not only time-frequency features, but also spatial relationships. Hence, we construct 3-dimensional depth radio images, instead of 2-dimensional ones. In this section, we will elaborate the signal processing steps, including the antenna selection, action segmentation, and high-dimensional radio image construction.

### 5.1. Antenna Selection

**Figure 2:**
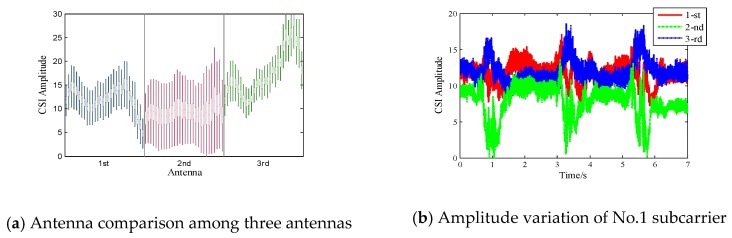
Antenna Selection.

Qian et al. [28] firstly demonstrated the aptness of tracking human path using two appropriately selected antennas for the dominant Doppler shift. As is shown in Figure 2a, the boxplot of 7-second CSI amplitude data across all 90 subcarriers reveals the distribution of CSI amplitude. We observe that the second antenna, with lower average amplitude values, is more likely to produce larger dynamic responses. This indicates that the second antenna has a weaker static component and is more likely to be distorted by micro-movements. Figure 2b depicts the amplitude variation of the No.1 subcarrier received in three different antennas. We can see the second antenna possesses the most significant amplitude fluctuations compared with the other two antennas. Inspired by the above two observations, we select the most sensitive antenna as the reference from the three receiving antennas and only leverage its amplitude information to detect motion transitions.

### 5.2. Gesture Segmentation

**Figure 3:**
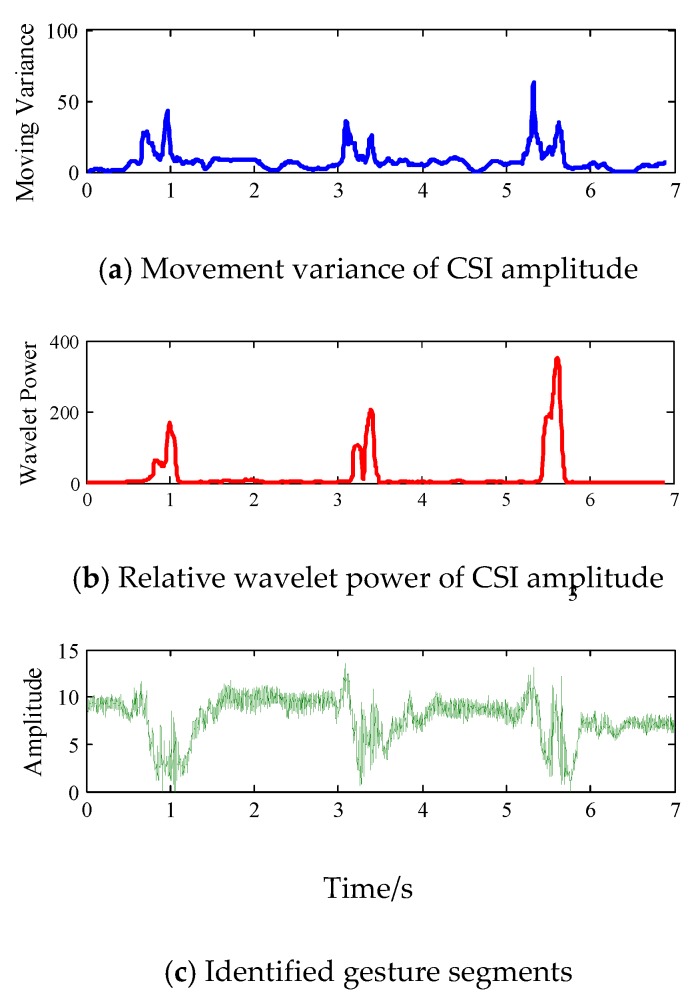
Illustration of motion detection and segmentation.

In reality, signal segmentation is an indispensable step for real-time gesture recognition, since it enables low computation cost and timely dynamics detection. For practical real-time gesture recognition, if we failed to detect the start points and end points, we would have to manually activate the system and deal with redundant information. Previous work [30] adopts moving variance or short time energy to indicate signal distortions because it accumulates the variations within a short time, as is shown in Figure 3a. Specifically, we request that one volunteer continuously performs three finger gestures. The participant performs each gesture using the following steps: raise the right hand, stretch fingers, retract fingers and put down the hand. Noting that movement variance could reflect the rough trend of signal variation caused by human part, however, the value is vulnerable due to unrelated motion interferences or background noises.

To detect subtle finger gesture transitions more efficiently, we develop a novel segmentation method based on an insight. Compared with the movement of other body parts, finger gestures produce slighter but relatively higher-frequency distortions. It is a natural choice to detect the transition points in specific frequency sub-bands. Therefore, we were inspired to adopt a wavelet energy-based segmentation method, which mainly consists of the following steps.

Firstly, we decompose raw CSI amplitude into approximation coefficients and detail coefficients. Generally, wavelet decomposition adopts a series of a low-pass filter *l*[*k*] and high-pass filter *h*[*k*] to process a raw signal with length *I*, producing both the approximation coefficients (AC) *A_m,l_* [*I*] and detail coefficients (DC) *A_m,h_* [*I*] of *m*-th level simultaneously as:

(4) $$A_{m,l}[I]=\sum_{k=0}^{K-1}A_{m-1,l}[2I-k]l[k],$$

(5) $$A_{m,h}[I]=\sum_{k=0}^{K-1}A_{m-1,h}[2I-k]h[k],$$

where AC depicts the fluctuation shape of CSI amplitude on a coarse scale (i.e., dominant body movements), while DC captures the fine details of signal dynamics (i.e., subtle finger gestures). We rebuild multi-scale signals on six levels with Daubechies db6 wavelet and only utilize DC to avoid the disturbance of irrelevant motions.

Then we resort to sliding windows to calculate wavelet power, since it strengthens the sensitivity by combing squared amplitude values in a short time. To strike a balance between time efficiency and accuracy, we set the window size *w* at 100, step width *s* at 50 and step number as *Q* = (*l − w*)/*s* + 1. We calculate the wavelet energy in the *m*-th level and the energy summation at the *q*-th step as:

(6) $$E_{m}[q]=A_{m}{}^{2}[q],E_{sum}[q]=\sum_{m=1}^{6}E_{m}[q]\;$$

Finally, we normalize the value as *p_m_*[*q*] = *E_m_*[*q*]/*E_sum_*[*q*], which represents the relative wavelet power, detecting the signal distortions of interest without additional noise reduction. Specifically, when we set the transmitting rate at 500 pkt/s, the gesture-corresponding frequency components mainly exist in the DCs of the 6th layer. In Figure 3b, we can see that relative wavelet power is more sensitive to subtle finger movements, since it efficiently excludes the disturbance of irrelevant body movements. Moreover, it is resistant to environmental noise, while movement variance can be more sensitive. Therefore, we only need to predefine a simple threshold by empirical study to indicate motion transitions. In Figure 3c, it is obvious that the most discriminative signal fluctuations induced by finger gestures are preserved, while the irrelevant body motions are discarded, which demonstrates the efficiency of our proposed segmentation method.

### 5.3. Image Construction

**Figure 4:**
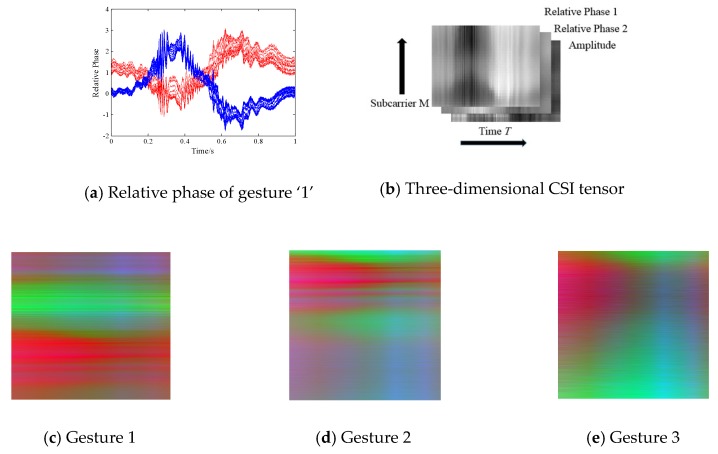
Image Construction.

Recall that we aim to construct discriminative 3-dimensional radio images by fully exploiting the amplitude and phase information. On the one hand, we have extracted sensitive amplitude without the loss of motion information. On the other hand, we have to cope with time-variant random phase noises caused by unsynchronized transmitters. In this paper, we refer to a novel phase sanitation method proposed in [41]. In particular, we apply conjugate multiplication among antenna pairs to remove the same CSI phase offsets across subcarriers:

(7) $$\tilde{H_{t}^{n}}=H_{t}^{n,m_{1}}{\left(H_{t}^{n,m_{2}}\right)}^{*}=\left|\tilde{H_{t}^{n}}\right|e^{j\tilde{\theta_{t}^{n}}},$$

where $\tilde{H_{t}^{n}}$, |$\tilde{H_{t}^{n}}$|, $\tilde{\theta_{t}^{n}}$ denotes the reconstructed conjugate matrix, amplitude and relative phase information. *H_t_^n,m^_1_* and *H_t_^n,m^_2_* are the *n*-th subcarrier measurements of the *m_1_* and *m_2_* antenna pair. We note that relative phase information could not only capture the micro *cm*-scale ($\Delta d=-\lambda \tilde{\theta_{t}^{n}}/2\pi$, wavelength λ is 12 cm) movement by removing static components, but also avoid the random phase offsets in phase measurements. We direct interested readers to Widance [41] for more detail. Figure 4a shows the extracted relative phase of all 30 subcarriers in the TX-RX_1 (red lines) and TX_RX_2 (blue lines) antenna pair when a volunteer performs finger gesture ‘1’. We observe that the relative phase could reveal obvious signal variations when finger gesture is performed, which indicates its sensitivity. Based on this observation, we are inspired to construct distinct 3-dimensional (channel) radio images based on one amplitude signal and two relative phase signals.

As is shown in Figure 4b, we design a novel sensitive CSI tensor consisting of two relative phase images and one amplitude image. Each image contains *M* × *T* pixels, where *M* denotes subcarrier indices from 1 to 30 (we interpolate the column value for finer granularity), *T* is the specific time slices. The higher the pixel value is, the darker the gray image. We mention that previous works only conduct the image processing method on a 2-dimensional matrix, which can be considered as a gray-scale map without color or spatial information. We creatively build 3-dimensional depth radio images for gestures 1, 2, and 3 with sensitive signal properties, and visualize them in the form of 3-channel RGB images in Figure 4c–e. As shown, the three radio images reveal distinctive color and texture characteristics. In addition, compared with existing gray-scale image processing, constructing CSI color images enables multi-scale features to be explored and exploited, which contributes to the detection of subtle finger gestures.

## 6. Image Processing

In this section, we transfer the problem from signal pattern recognition to color image classification and design a series of novel image processing techniques accordingly. (1) To jointly reduce image noise, HOSVD-based de-noising is adopted to extract high-dimensional principal components; (2) To lower the computation cost and regularize the size of the radio image, we implement the concept of *Selective Search* and select a characteristic 500 × 500 region based on color and texture features. (3) To achieve efficient image classification, we resort to a 7-layer CNN architecture without extensive parameter study and SoftMax function to output classified labels.

### 6.1. HOSVD-Based Image De-Noising

**Figure 5:**
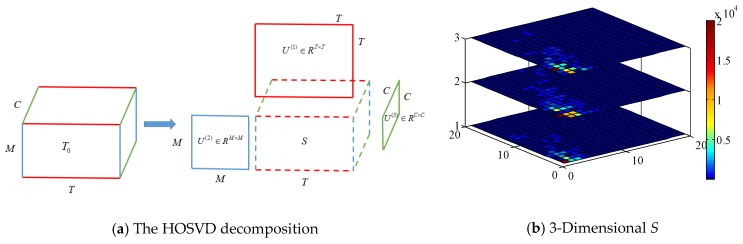
HOSVD-based Image De-noising.

Bearing in mind that a color radio image is constructed with multi-dimensional relations, we aim to reduce irrelevant noises left over by fully exploiting tensor properties. HOSVD is an efficient tool to extract high-dimensional core component via performing tensor factorization, which is an extension of matrix SVD. The basic idea of HOSVD is to select the dominant eigenvalues by nullification of individual values below a predefined threshold [42]. Let us denote radio image as *T_0_*∈*R^M^*^×*T*×*C*^, *C* is the channel number. The HOSVD process of tensor *T_0_* is depicted in Figure 5a, and its decomposition formula can be written as:

(8) $$T_{0}=S\times_{1}U^{\left(1\right)}\times_{2}U^{\left(2\right)}\times_{3}U^{\left(3\right)},$$

where *U*^(1)^∈*R^T^*^×*T*^, *U*^(2)^∈*R^M^*^×*M*^, *U^(^*^3)^∈*R^C^*^×*C*^ are orthogonal matrices, *S*∈*R^M^*^×*T*×*C*^ is the 3-dimensional coefficient array, ×_1,2,3_ is the 1st, 2nd and 3rd model tensor product, respectively.

Compared with applying SVD in each matrix separately, HOSVD takes advantage of relations between orders by reordering the elements of an *N*-mode array into a matrix (e.g., a 3 × 4 × 5 tensor can be arranged as a 12 × 5 matrix or a 3 × 20 matrix). Figure 5b plots the 3-dimensional eigenvalues of core tensor *S* when a volunteer performs gestures. X-axis and Y-axis denote the selected number of the eigenvalue matrix, Z-axis is spatial dimension, and eigenvalue magnitude is represented by color-map. We observe that significant singular values only concentrate on a few samples with small indices, which exponentially overwhelm other eigenvalues and almost capture the whole image information. Therefore, the task of noise removal can be achieved by applying a hard threshold δ to reconstruct core tensor $\tilde{S}$: if *S*(*M,T,C*) *≤ µ*, then $\tilde{S}$(*M*,*T*,*C*) = 0; only when *S*(*M*,*T*,*C*) ≥ *µ*, $\tilde{S}$(*M*,*T*,*C*) = *S*(*M*,*T*,*C*), *µ* is set at the 90 centile of 1-D matrix eigenvalues. In this way, we could rebuild $\tilde{T}$ via formula 8, with dominant eigenvalue coefficients reserved and slight singular value discarded. Though simple, it avoids the problem of ‘out of memory’ and multi-rank parameter estimation when we manipulate a large tensor with MATLAB *tensor toolbox*, thus realizing fast computation [43].

### 6.2. Image Region Selection

At the very beginning, we survey the related state of the art and illustrate the rationality of image region selection. As CNN-based deep learning methods have led to revolutionary change in the visual community and substantially improved classification performance, there is still a critical issue that needs to be considered: the fixed-size constraint of the CNN network. To be more specific, the fully connected layer at a deeper stage of existing CNN network requires a fixed length of input by definition [44]. Simple cropping or warping may lose the most discriminative features and induce unwanted geometric distortion. *SPP-net* [39] feeds images with slightly varying scales during training and generates fixed-length representations by adding a *spatial pyramid pooling* (SPP) layer, while the size of radio images can be more arbitrary because gesture duration varies. We borrow the idea from the *Selective Search* [45] and propose a novel region selection method based on weighted color and texture features. Instead of grouping initial regions with multiple similarity strategies until the whole image becomes a single region, we design a sliding region method by comparing the color features of fixed-size region with weighted values and choosing the most significant one.

Specifically, we then extract color moment *mean*, *standard deviation*, and *skewness* to depict the color distribution of each matrix, to capture gray-level texture features from gray-level pairs and weighted EMD-based values to show the similarity between matrices. Let *p_i,j_^k^* denote the [*i*,*j*]-th pixel value of the *k*-th channel, the fixed size of the region is *N* × *N*, and color moment features can be calculated by the following formulas:

(9) $$\begin{array}{rr} \mathrm{Mean}: & E^{k}=\sum_{j=1}^{N}\sum_{i=1}^{N}\frac{1}{N^{2}}p_{{}_{ij}}^{k}, \end{array}$$

(10) $$\begin{array}{rr} \mathrm{Standard}\;\mathrm{Deviation}: & \sigma^{k}=\frac{1}{N}\sqrt{\sum_{j=1}^{N}\sum_{i=1}^{N}{\left(p_{{}_{ij}}^{k}-E^{k}\right)}^{2}}, \end{array}$$

(11) $$\begin{array}{rr} \mathrm{Skewness}: & s^{k}=\sqrt[{3}]{\frac{1}{N^{2}}\sum_{j=1}^{N}\sum_{i=1}^{N}{\left(p_{ij}^{k}-E^{k}\right)}^{3}}, \end{array}$$

where $E^{k}$, $\sigma^{k}$, and $s^{k}$ represent the average signal intensity, magnitude variation level and how asymmetric the distribution is, respectively.

To further calculate the spatial relations of similar gray tones, we adopt gray-level co-occurrence matrices, which are easy to compute, and extract four standard features from *Ng* distinct gray levels, which can be expressed as:

(12) $$\begin{array}{rr} \mathrm{Angular}\;2\mathrm{nd}\;\mathrm{Moment}: & ATW^{k}=\sum_{j=1}^{Ng}\sum_{i=1}^{Ng}(p_{ij}^{k}{)}^{2}, \end{array}$$

(13) $$\begin{array}{rrr} \mathrm{Contrast}: & Con^{k}=\sum_{j=1}^{Ng}\sum_{i=1}^{Ng}d^{2}p_{ij}^{k}, & \mathrm{where},\left|i-j\right|=d\; \end{array}$$

(14) $$\begin{array}{rr} \mathrm{Correlation}: & Cor^{k}=\frac{\sum_{j=1}^{Ng}\sum_{i=1}^{Ng}(ij)p_{ij}^{k}-\mu_{x}\mu_{y}}{\varepsilon_{x}\varepsilon_{y}}, \end{array}$$

(15) $$\begin{array}{rr} \mathrm{Entropy}: & Ent^{k}=-\sum_{j=1}^{Ng}\sum_{i=1}^{Ng}p_{ij}^{k}\ln(p_{ij}^{k}), \end{array}$$

where *ATW^k^* illustrates the level of homogeneity of *k*-channel matrix, *Con^k^* measures the difference in luminance, *Cor^k^* indicates the linear level between pix pairs and *Ent^k^* quantifies the disorder, randomness and complexity of the proposed region.

**Figure 6:**
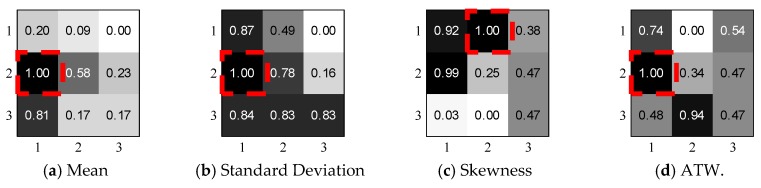

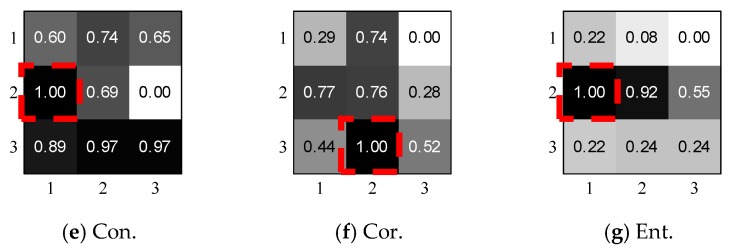
Experiment Study by Comparing Color and Texture Features in Different Regions.

To demonstrate the aptness of three color features and four texture features, we conduct a simple experimental study by applying a 500 × 500 sliding region with step size 250 through the whole 1000 × 1000 image and extract 7 × 3 features from a total of nine segmented regions. To eliminate the feature dimensions, we normalize the feature matrix and aggregate the same feature of the same three regions. In Figure 6, we notice that different features show varied performance in different regions, revealing the image properties from a different perspective, while the (1, 2) region contains the maximum valuable features which are marked with red frames. Inspired by the above observations, we design a novel indicator which combines all color and texture features:

(16) $$\begin{array}{l} Score(A,B)=a_{1}E_{A,B}+a_{2}\sigma_{A,B}+a_{3}s_{A,B}+\\ a_{4}ATW_{A,B}+a_{5}Con_{A,B}+a_{6}Cor_{A,B}+a_{7}Ent_{A,B} \end{array},$$

where *a_1_*,…, *a_7_* denotes the descending order number of all regional features. For example, the (1, 2) region gets the largest *mean* value in all 9 regions, so *a_1_* is allocated with the highest weighted value 9, *a_2_* receives the second-highest value 8, vice versa. In real experiments, we set region size at 500 × 500 and step size at 100 for computation efficiency. Additionally, we select the top ten image regions in the training step to improve the tolerance of image distortion and only choose the region with the highest weighted score. Therefore, we could relieve the constraints of radio image size.

### 6.3. CNN-Based Classification

**Figure 7:**
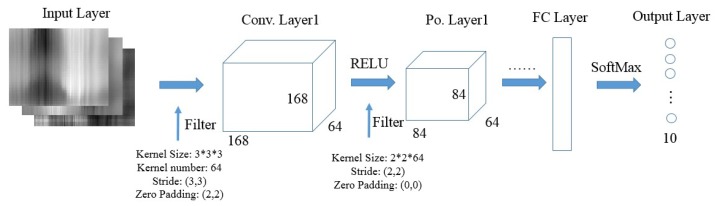
CNN Architecture.

We adopt a 7-layer CNN to recognize input image regions and output classified labels. The CNN model possesses several attractive qualities over the previous DNN model. Firstly, CNN has much fewer connections and parameters, so it could facilitate computing speed. Secondly, CNN enables large-scale high-dimension data training with current GPUs and local fields of perception. Thirdly, CNN could automatically capture abstract pixel-level characteristics through a hierarchical architecture with highly optimized implementation of convolution filters. Therefore, we believe CNN theoretically outperforms the DNN model in image classification. Figure 7 shows the architecture and related parameters of our CNN model, which consists of three core building blocks: Input Layer, Convolution (Conv.) Layer, Pooling (Po.) Layer, and Fully Connected (FC) Layer. We will introduce the function of each part and provide some key parameters.

*Input Layer*. For each gesture, we input the top 10 labeled fixed-size images (i.e., 500 × 500 × 3) to train the CNN model and only use the top 1 region in the predicting step. Therefore, input layer will keep the size of an image with width 500, height 500 and depth 3.

*Conv. Layer*. Conv. Layer has natural advantages by applying a set of learnable spatial filters to achieve local connectivity and parameter sharing, because it connects each neuron to only a local region of the input volume (local in space, but all along the entire depth). Additionally, each filter slides through the whole input volume with the same kernel and generates 3-dimensional abstract feature maps that give the responses in every spatial position. For example, if we decide to adopt 64 3 × 3 × 3 convolutional kernels (or the receptive field) to filter a 500 × 500 × 3 raw image (stride length is 3 and zero padding size is 2), then we would obtain a volume of 168 × 168 × 64. Assuming that we use a total of 168 × 168 × 64 neurons, here, and only [(3 × 3 + 1) × 64] × 168 × 168 = 18,063,360 connections, the calculated amount in the FC layer can be explosive, since the number of connection is (500 × 500) × (168 × 168 × 64) ≈ 4.5 × 10^11^.

In our experiment, we summarize some tricks to select appropriate parameters. First, we set the kernel size as 3 × 3 × 3, since stacked small kernels provide finer characteristics [46]. Secondly, we use stride 3 in the first two layers to reduce the dimension and stride 1 in the last two layers for feature traversal. Thirdly, we put zeros on the image border to allow an exact division. Finally, we tune the number of kernels to learn sufficient characteristics and control the output number. We also add Rectified Linear Unit (RELU) layers between Conv. Layers and Po. Layers so as to activate the positive part of feature maps.

*Po. Layer*. The function of pooling layer is to perform a down-sampling operation vertically and horizontally through the whole feature map. We adopt max pooling function to reveal only the maximum value of each cluster in the prior layer. In this way, we gain computation efficiency by obtaining less spatial information and avoid over-fit by training fewer parameters. Since there is no weight to train, we set the kernel size and stride size as (2, 2) through the whole process.

*FC Layer*. Fully connected layer connects every neuron in the former Po. Layer to every neuron on the hidden layer, which aims to combines all deep features and train the output after all the Conv. Layer and Po. Layer. The output size of FC layer is designed to be 10 in order to align the corresponding gesture labels. A softmax regression layer is added on the bottom of the architecture, which serves to squash the 10-dimensional feature data to the probability $p_{j}$ of *j* clusters, which is given by

(17) $$\begin{array}{ll} p_{j}=\frac{e^{z_{j}}}{\sum_{k=1}^{z_{j}}e^{z_{k}}}, & \mathrm{for}\;j=1\ldots ,10, \end{array}$$

we adopt cross entropy as a loss function to compare the difference between the predicted labels and true labels, and further utilize the Stochastic Gradient Descent with Momentum to train the parameters.

## 7. Experimental Study

In this section, we firstly introduce the experimental configuration, then describe the performance evaluation, and finally discuss the impacts of the experimental variables and limitations.

### 7.1. Experiment Configuration

**Hardware setup**. We implement *DeepNum* using a pair of ThinkPad X200 laptops (Lenovo, Beijing, China) equipped with modified Intel 5300 NICs (Intel, Santa Clara, USA) and installed *CSITool* [26]. Both laptops are set up to work in monitor mode on Channel 165 at 5.825 Hz, one acts as the transmitter with only one external antenna, the other one serves as the receiver, possessing three external antennas for extra spatial information. Three receiving antennas are linearly arranged with a spacing distance of 5.2 cm. The pack transmitting rate is set to 500 Hz for fast dynamics, and transmission power is set to 15 *dBm* by default. During the training process, we employ *MATLAB 2017b* (The MathWorks, Natick, USA) running on an Intel i7-5700HQ 2.70GHz CPU (Intel, Santa Clara, USA) to process the CSI matrix.

**Figure 8:**
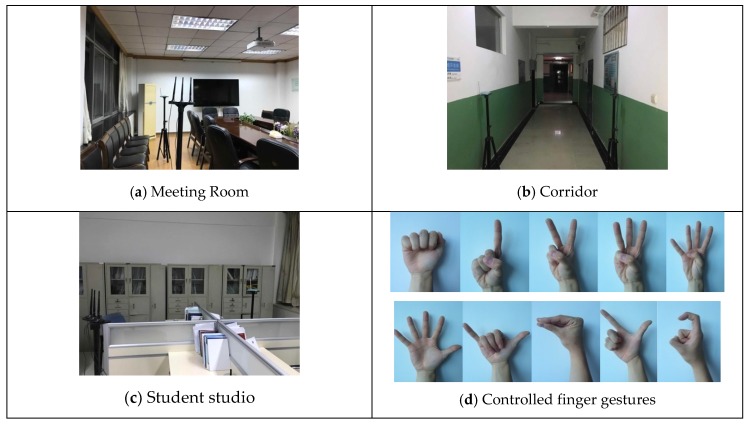
Experimental setup.

**Environment and data collection**. All experiments are conducted in typical indoor scenarios of an academic building, including a meeting room, a corridor and a student studio. As is shown in Figure 8a–c, there is a 6 × 6 m^2^ meeting room with a conference table and twelve chairs, a 2 × 20 m^2^ empty corridor surrounded by walls and a 6 × 12 m^2^ complex student office with two worktables, and multiple blocks and electronic interferences. We set the distance between laptops as 2 m, 1 m and 3 m, respectively, and fix the height of the antennas at 1.3 m. Eight male and two female student volunteers perform controlled finger gestures as illustrated in Figure 8d. All volunteers major in computer engineering, and have ages between 22 and 35. When CSI collection begins, the participant is asked to follow the instructions given by a recorder, step into the wireless environment and arrive at appointed locations. Then the recorder randomly gives the command number from 0 to 9 and records the digits, while the participant continuously performs the finger gestures with the body stationary. During the experiments, every participant performs each gesture by following similar steps: raise the right hand, stretch fingers, retract fingers and put down the hand. We collect a total of 300 instances for all 10 volunteers in all 3 scenarios within a 3-week period. Each instance contains 20 action segments covering all 10 digit gestures with short breaks. In the training stage, we use 50% of the records for all 10 volunteers to train the CNN model and the remaining 50% of the records to test the performance.

**Evaluation metrics.** To evaluate the system performance, we adopt two common metrics. (1) *Confusion matrix*. The x-axis and y-axis represent the classified finger gestures and actually performed finger gestures, respectively. The number in the (*i*, *j*)-th cell denotes the probability of the *j*-th gesture classified into the *i*-th label. (2) *Accuracy*. We conduct comparison studies and parameter studies to verify the system robustness.

### 7.2. Performance Evaluation

**Figure 9:**
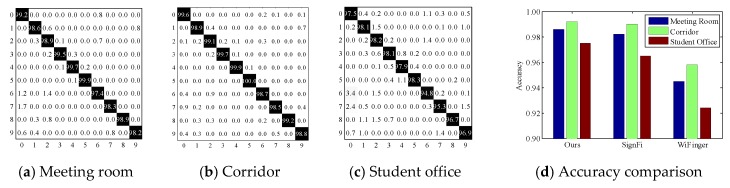
Performance evaluation.

**Overall performance**. We first report the overall performance of *DeepNum*. As is shown in Figure 9a–b, *DeepNum* yields a considerable average accuracy of 98% and 99% in the empty meeting room and corridor. In particular, all gesture accuracy is improved in the corridor because of the shorter transmitting distance and the stronger reflected signals. The average accuracy is 2% lower in the student office (Figure 9c) due to the multiple blocks and electronic interferences. We also notice a similar distribution of prediction accuracy in all three environments. For instance, digit ‘5’ dominates in the confusion matrix of all experimental scenarios because it requires a full stretch of all 5 fingers and induces larger signal fluctuations, while digit ‘6’ is easily confused with ‘0’ and ‘2’, since they have similar movement patterns.

**Compared to baselines**. We survey the state-of-the-art Wi-Fi-based gesture recognition systems from the last 3 years and list the relevant works in Table 1. To fully demonstrate the high performance of *DeepNum*, we implement *DeepNum* alongside two typical schemes, *SignFi* [23] and *WiFinger* [13], for comparison. The reason is two-fold. On the one hand, as the emerging masterpiece in using a deep learning model to recognize finger gestures, *SignFi* imports full amplitude and phase information into a typical CNN model, and achieves high accuracy in specific scenes. However, it circumvents the selection of raw CSI signals and the signal segmentation step. We aim to show the efficiency of the image construction step and the feasibility of the region selection step by comparison. On the other hand, since most of works follow a similar methodology (i.e., handcrafted feature extraction and traditional classification algorithms), we choose *WiFinger* [13] as a benchmark in order to show the superiority of the deep learning model. In Figure 9d, we observe that *WiFinger* has an acceptable overall accuracy of over 93% in all three scenarios; however, it is lower than the two CNN-based schemes. Furthermore, *WiFinger* fails to consider both amplitude and phase information, which may miss informative signal features. *SignFi* achieves competitive results in both the meeting room and corridor by careful parameter tuning. However, the accuracy drops more obviously in the student office. This is mainly because motion-induced distortions are overwhelmed by background noises. Thanks to sensitive image construction and sanitation, *DeepNum* outperforms *SignFi* in NLOS scenarios. Moreover, *DeepNum* avoids the size requirement, which renders it more practical.

### 7.3. Parameter Study

**Figure 10:**
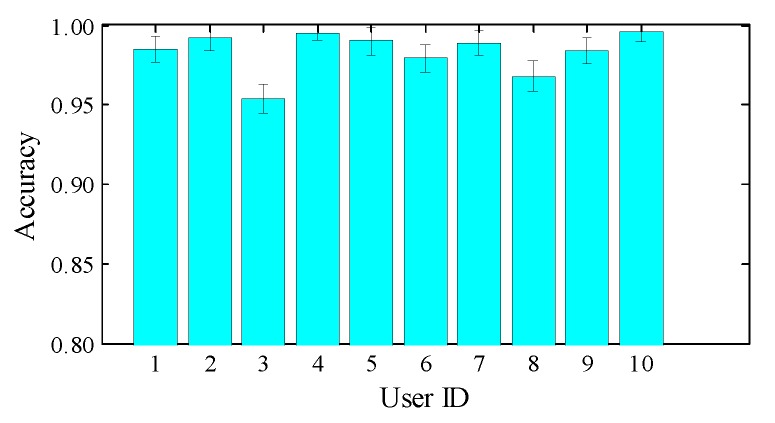
Impact of user diversity.

**Impact of user diversity.** To evaluate whether our system works consistently for different users, we employ 10 volunteers of different genders, heights, weights and body mass index (BMI). Table 2 shows the statistics of the participants; User ID 1 to 8 are male, and User ID 9 to 10 are female. We also present the recognition results of all participants in Figure 10. As shown, *DeepNum* is able to handle diversity and achieve robust recognition for all individuals with an accuracy higher than 95%. However, there is no direct correlation between user diversity and accuracy. We leave further study of this for our future work.

**Figure 11:**
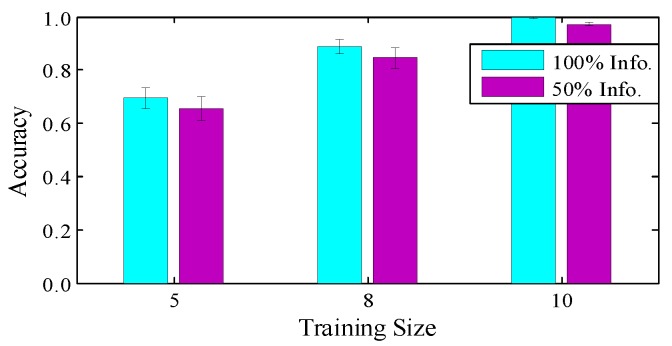
Impact of training strategy.

**Impact of training strategy.** In the experiments, we collected a total of 6000 gesture segments from all 10 volunteers. Specifically, we use 50% of the records of all 10 volunteers to train the CNN model, and the remaining 50% of the records to test the performance. To further discuss the impact of the training strategy, we train the CNN model with decreased numbers of users and numbers of training samples per user. In Figure 11, we firstly train the model with all records and test the performance using the data of one user, which obtains the highest accuracy. Our training method uses fewer training samples but achieves considerable results, with an accuracy of 98%. We also decrease the number of users from 10 to 5, and use the remainder as the test groups. A significant drop is observed in the accuracy from 98% to 65%, while the differences between diverse training sample sizes is not significant. So adding participant diversity really matters for a practical system.

**Figure 12:**
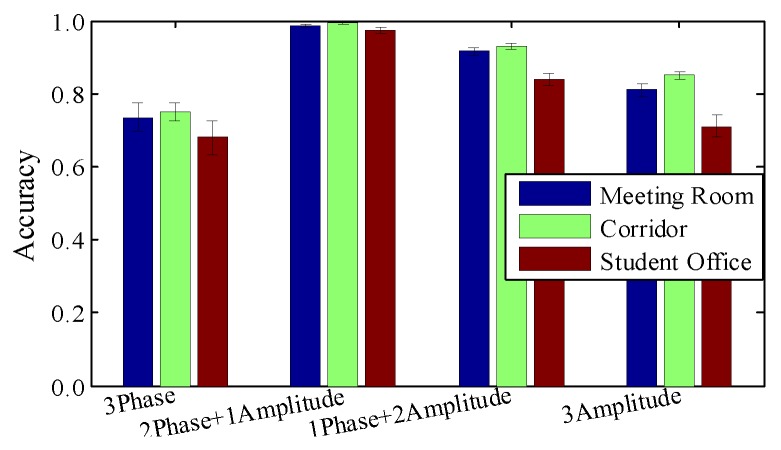
Impact of raw material.

**Impact of raw material**. To evaluate the efficiency of image construction, we perform contrast experiment by applying various combinations of amplitude and relative phases. Please note that the depth of the radio image is fixed at 3, since that is a requirement of the current CNN model. We leave ‘higher-dimensional tensor processing’ for future work. Figure 12 shows that amplitude or relative phase information alone are not sufficient to detect slight finger gestures. Based on observation, we extract sensitive amplitudes from one antenna and two relative phases from all three antenna pairs.

**Figure 13:**
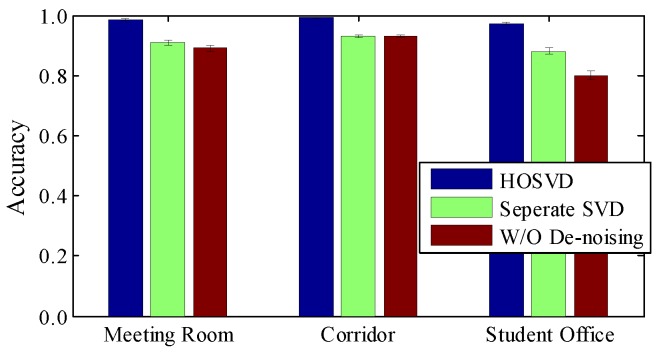
Impact of image de-noising.

**Impact of image de-noising**. We also check the influence of HOSVD, which reduces the noise in the radio image. In Figure 13, without any de-noising step, the average accuracies are 83%, 85% 78% for the meeting room, corridor and student office, respectively. This implies that the radio image is built up from sensitive and robust information. Applying SVD in each matrix helps a little in overall accuracy, but if we perform HOSVD from a high-dimensional perspective, the accuracy can be improved significantly, especially in NLOS environments. The reason for this is that HOSVD is able to manipulate multi-channel correlation simultaneously, so slight motion-induced distortions can be captured even in a noisy environment.

**Figure 14:**
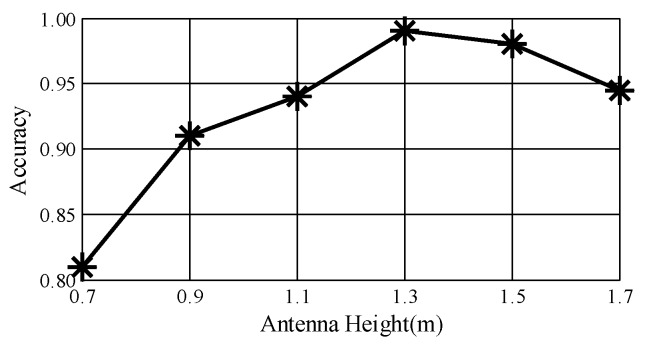
Impact of antenna height.

**Impact of antenna height**. To determine the perfect height of the antenna pairs, we conduct experiments at heights of 0.7 m, 0.9 m, 1.1 m, 1.3 m, 1.5 m and 1.7 m. Figure 14 plots the performance of *DeepNum*. Under the initial condition, *DeepNum* has its lowest average accuracy of 81%, due to irrelevant body movements and severe multi-path effect. The accuracy climbs to a peak as the antenna height reaches 1.3 m, with a strong reflected signal. We also notice that *DeepNum* consistently yields high accuracy at a height of 1.7 m. Therefore, *DeepNum* could work well in real indoor environments, since most Wi-Fi devices are placed on the ceiling.

**Figure 15:**
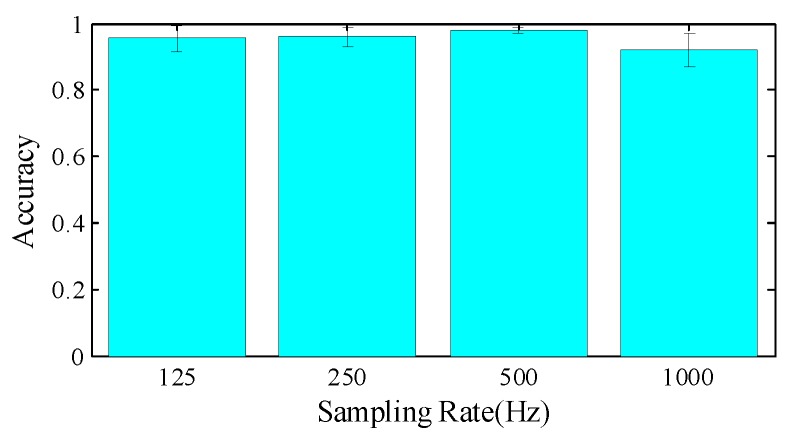
Impact of sampling rate.

**Impact of sampling rate**. Please note that we extract image regions with a fixed size of 500 × 500; the high sampling rate introduces fine-grained local movement information, but limited global gesture features. To strike a balance, we first initialize the sampling rate at 1000 Hz and gradually discard CSI data to obtain sampling rates of 500 Hz, 250 Hz and 125 Hz. In Figure 15, we note that 500 Hz achieves the best performance, since it includes 1 s gesture information with sufficiently detailed features. A sampling rate of 1000 Hz or higher may lower the performance, because the image region contains less temporal or gestural contextual information.

**Impact of feature extraction**. We further conduct experiments and compare the accuracy of different feature extraction strategies in Table 3. As the size requirement really matters, we firstly select the fixed-size regions in the center of images, without feature extraction step. The results are unacceptable, since the regions only preserve a part of the motion-induced information. Using color features or texture features as a priori knowledge significantly improves the accuracy in the meeting room and corridor, while the performance could be affected by multiple interferences in the student office, at 78.9% and 83.4%, respectively. We utilize both color and texture features so as to enhance the robustness of region selection, and achieve the highest accuracy in the three scenes.

### 7.4. Limitations and Discussion

**Multiple moving objects.** Targeting single-hand finger gesture recognition of single user, *DeepNum* is vulnerable to multiple moving hands or bodies, which may induce super-imposed signal fluctuations at the receivers and dampen the promotion of gesture-based applications. Wang et al. [47] propose a multi-person breath monitoring method based on tensor decomposition with a pair of Wi-Fi devices. However, this requires prior knowledge of the number of users or needs to be tackled as a NP-hard problem. How to recognize finger gestures performed by multiple hands simultaneously will still remain an open and challenging problem for the future.

**Increasing sample diversity**. User diversity and training strategy are important factors for ubiquitous gesture recognition. In our experiments, we try to increase sample diversity by recruiting different kinds of volunteers; however, it is labor-intensive to collect such an amount of CSI data. *WiAG* [15] offers an alternative by applying translation functions on limited training samples, which inspires us to broaden sample diversity and increase training samples by slight parameter adjustment.

**Designing proper architecture**. In this paper, since our major concern is to break the constraints of size requirements, we simplify the CNN architecture by adopting a conventional 7-layer CNN model and adjusting its related parameters (i.e., kernel size, kernel number, stride size and zero padding) for considerable results. In the future, we will seek to promote system performance in NLOS environments by deepening the model and designing more appropriate architectures based on up-to-date deep learning models, like VGG-net [46], GoogleNet [48] and Deep Residual Learning [49].

**Tracking long-term behaviors.** Borrowing the idea from the image-based domain, *DeepNum* recognizes finger gestures from a high-dimensional perspective and extracts deeper features based on the CNN model, which can be applied to various applications. However, we argue that CNN is not a panacea for all scenarios, especially with respect to long-term behavior monitoring, such as habit modeling and health monitoring. As an analogy, these applications seem more like video-based problems rather than image-based issues, and require a whole different line of thinking and context awareness. Please note that CNN cannot capture the relations between temporal segments, and we leave the behavior modeling for our future work.

**Computation cost.** To evaluate the computation cost of *DeepNum*, we calculate the average running time on a laptop with an Intel i7-5700HQ 2.70GHz CPU (Intel, Santa Clara, USA). Table 4 illustrates the running time of each processing part. As shown, the major time cost comes from the region selection step. This is because we select the most significant region by extensive search. The calculation time can be reduced by using high-performance processors. Since the total running time for each gesture is only 57.85 ms, it is still acceptable for Wi-Fi-based systems, which will generally achieve finger gesture recognition on the scale of seconds.

## 8. Conclusions

In this paper, we present a novel finger gesture recognition system, named *DeepNum*, the core part of which is radio image construction and image manipulation. In the first part, we extract amplitude from the most sensitive antenna and segment action slices using wavelet energy. To fully exploit signal characteristics, we obtain two sensitive relative phase matrices from pairwise coupling antenna pairs and further construct a 3-dimensional tensor. Next, in the second part, we resort to HOSVD for image de-noising and region selection for the input requirement of CNN model, which contributes to extracting deep features automatically and outputs classified results accurately. Experimental results show that *DeepNum* achieves an overall recognition accuracy of 98% in three typical indoor environments. From micro-movement detection to micro-movement recognition, and from statistical properties calculation to deep feature extraction, *DeepNum* goes a step further by processing CSI data from a higher-dimensional perspective. As a pioneer, *DeepNum* is expected to promote the progress of wireless sensing applications.

## Figures and Tables

**Table 1:** A comparison of state-of-the-art works for Wi-Fi-based gesture recognition.

Properties	Year	Granularity	Signal	Segmentation?	Classification Algorithm	Accuracy
*WiDraw* [11]	2015	Hand	Amp	×	×	91%
*WiKey* [12]	2015	Finger	Amp	√	DTW	93.5%
***WiFinger*** [13]	2016	Finger	Amp	√	KNN + DTW	90.4%
*WiFinger* * [14]	2016	Finger	Amp	√	DTW	93%
*DeNum* [21]	2016	Finger	Amp	×	DNN	94%
*WiAG* [15]	2017	Hand	Amp	√	KNN	91.4%
Wang J. et al. [20]	2017	Body	Amp + Phase	×	DNN	90%
Wang X. et al. [37]	2017	Body	Amp + AOA	×	CNN	87%
***SignFi*** [23]	2018	Finger	Amp + Phase	×	CNN	98%
***DeepNum***	2018	Finger	Amp + Phase	**√**	CNN	98%

**Table 2:** Statistics of participants.

User ID	Height/Weight (cm/kg)	BMI	User ID	Height/Weight (cm/kg)	BMI
1	179/79	24.7	6	176/75	24.2
2	182/68	20.5	7	172/55	18.6
3	180/70	21.6	8	181/68	20.8
4	177/65	20.8	9	167/52	18.7
5	183/73	21.8	10	165/55	20.2

**Table 3:** Accuracy of *DeepNum* using different features.

Experiments	Features	W/O	Color	Texture	Ours
Meeting Room	Accuracy (%)	58.5	84.5	86.3	98.6
Corridor	Accuracy (%)	63.3	90.1	92.7	99.2
Student Office	Accuracy (%)	51.8	78.9	83.4	97.5

**Table 4:** Running time of processing parts per gesture.

Parts	Signal Processing	Image De-Noising	Region Selection	CNN Classification	Total
Times (ms)	5.26	10.52	25.33	16.74	57.85

## References

[1] Rautaray S.S., Agrawal A. (2015). Vision based hand gesture recognition for human computer interaction: A survey. Artif. Intell. Rev..

[2] Panwar M., Mehra P.S. Hand gesture recognition for human computer interaction. Proceedings of the 2011 International Conference on Image Information Processing.

[3] Xu C., Pathak P.H., Mohapatra P. Finger-writing with smartwatch: A case for finger and hand gesture recognition using smartwatch. Proceedings of the 16th International Workshop on Mobile Computing Systems and Applications.

[4] Palacios J.M., Sagüés C., Montijano E., Llorente S. (2013). Human-computer interaction based on hand gestures using RGB-D sensors. Sensors.

[5] Sun C., Zhang T., Xu C. (2015). Latent support vector machine modeling for sign language recognition with Kinect. ACM Trans. Intell. Syst. Technol. (TIST).

[6] Funasaka M., Ishikawa Y., Takata M., Joe K. Sign language recognition using leap motion controller. Proceedings of the International Conference on Parallel and Distributed Processing Techniques and Applications (PDPTA).

[7] Pu Q., Gupta S., Gollakota S., Patel S. Whole-home gesture recognition using wireless signals. Proceedings of the 19th annual international conference on Mobile computing & networking.

[8] Kellogg B., Talla V., Gollakota S. Bringing gesture recognition to all devices. Proceedings of the 11th USENIX Conference on Networked Systems Design and Implementation.

[9] Wang J., Vasisht D., Katabi D. RF-IDraw: Virtual touch screen in the air using RF signals. Proceedings of the 2014 ACM Conference on SIGCOMM.

[10] Abdelnasser H., Youssef M., Harras K. Wigest: A ubiquitous WiFi-based gesture recognition system. Proceedings of the 2015 IEEE Conference on Computer Communications (INFOCOM).

[11] Sun L., Sen S., Koutsonikolas D., Kim K. Widraw: Enabling hands-free drawing in the air on commodity wifi devices. Proceedings of the 21st Annual International Conference on Mobile Computing and Networking.

[12] Ali K., Liu A.X., Wang W., Shahzad M. Keystroke recognition using Wi-Fi signals. Proceedings of the 21st Annual International Conference on Mobile Computing and Networking.

[13] Li H., Yang W., Wang J., Xu Y., Huang L. WiFinger: Talk to your smart devices with finger-grained gesture. Proceedings of the 2016 ACM International Joint Conference on Pervasive and Ubiquitous Computing.

[14] Tan S., Yang J. WiFinger: Leveraging commodity Wi-Fi for fine-grained finger gesture recognition. Proceedings of the 17th ACM International Symposium on Mobile Ad Hoc Networking and Computing.

[15] Virmani A., Shahzad M. Position and orientation agnostic gesture recognition usingWi-Fi. Proceedings of the 15th Annual International Conference on Mobile Systems, Applications, and Services.

[16] Wang T., Wen C., Wang H., Gao F., Jiang T., Jin S. (2017). Deep learning for wireless physical layer: Opportunities and challenges. China Commun..

[17] Wang X., Gao L., Mao S., Pandey S. (2017). CSI-based fingerprinting for indoor localization: A deep learning approach. IEEE Trans. Veh. Technol..

[18] Zhang W., Liu K., Zhang W., Zhang Y., Gu J. (2016). Deep neural networks for wireless localization in indoor and outdoor environments. Neurocomputing.

[19] Shi C., Liu J., Liu H., Chen Y. Smart user authentication through actuation of daily activities leveraging WiFi-enabled IoT. Proceedings of the 18th ACM International Symposium on Mobile Ad Hoc Networking and Computing.

[20] Gao Q., Wang J., Ma X., Feng X., Wang H. (2017). CSI-Based Device-Free Wireless Localization and Activity Recognition Using Radio Image Features. IEEE Trans. Veh. Technol..

[21] Bengio Y. (2009). Learning deep architectures for AI. Found. Trends Mach. Learn..

[22] Zhou Q., Xing J., Li J., Yang Q. A device-free number gesture recognition approach based on deep learning. Proceedings of the 2016 12th International Conference on Computational Intelligence and Security (CIS).

[23] Ma Y., Zhou G., Wang S., Zhao H., Jung W. (2018). SignFi: Sign Language Recognition Using WiFi. Proc. ACM Interact. Mob. Wearable Ubiquitous Technol..

[24] Molchanov P., Gupta S., Kim K., Pulli K. Multi-sensor system for driver’s hand-gesture recognition. Proceedings of the 11th IEEE International Conference and Workshops on Automatic Face and Gesture Recognition (FG).

[25] Lien J., Gillian N., Karagozler M.E., Amihood P., Schwesig C., Olson E., Poupyrev I. (2016). Soli: Ubiquitous gesture sensing with millimeter wave radar. ACM Trans. Graph. (TOG).

[26] Halperin D., Hu W., Sheth A., Wetherall D. (2010). Predictable 802.11 packet delivery from wireless channel measurements. ACM SIGCOMM Comput. Commun. Rev..

[27] Xie Y., Li Z., Li M. (2018). Precise power delay profiling with commodity Wi-Fi. IEEE Trans. Mob. Comput..

[28] Qian K., Wu C., Yang Z., Liu Y., Jamieson K. Widar: Decimeter-level passive tracking via velocity monitoring with commodity Wi-Fi. Proceedings of the 18th ACM International Symposium on Mobile Ad Hoc Networking and Computing.

[29] Qian K., Wu C., Zhang Y., Zhang G., Yang Z., Liu Y. Widar 2.0: Passive human tracking with a single wi-fi link. Proceedings of the ACM MobiSys, Athen.

[30] Wang H., Zhang D., Wang Y., Ma J., Wang Y., Li S. (2017). RT-Fall: A Real-Time and Contactless Fall Detection System with Commodity WiFi Devices. IEEE Trans. Mob. Comput..

[31] Wang W., Liu A.X., Shahzad M. Gait recognition using wifi signals. Proceedings of the 2016 ACM International Joint Conference on Pervasive and Ubiquitous Computing.

[32] Guo X., Liu B., Shi C., Liu H., Chen Y., Chuah M. WiFi-Enabled Smart Human Dynamics Monitoring. Proceedings of the 15th ACM Conference on Embedded Network Sensor Systems.

[33] Hinton G.E., Salakhutdinov R.R. (2006). Reducing the dimensionality of data with neural networks. Science.

[34] Krizhevsky A., Sutskever I., Hinton G.E. Imagenet classification with deep convolutional neural networks. Proceedings of the Advances in Neural Information Processing Systems.

[35] Dahl G.E., Yu D., Deng L., Acero A. (2012). Context-dependent pre-trained deep neural networks for large-vocabulary speech recognition. IEEE Trans. Audio Speech Lang. Process..

[36] Liu C., Zhang L., Liu Z., Liu K., Li X., Liu Y. Lasagna: Towards deep hierarchical understanding and searching over mobile sensing data. Proceedings of the 22nd Annual International Conference on Mobile Computing and Networking.

[37] Wang X., Wang X., Mao S. CiFi: Deep convolutional neural networks for indoor localization with 5 GHz Wi-Fi. Proceedings of the 2017 IEEE International Conference on Communications (ICC).

[38] Lv S., Lu Y., Dong M., Wang X., Dou Y., Zhuang W. (2017). Qualitative action recognition by wireless radio signals in human–machine systems. IEEE Trans. Hum. Mach. Syst..

[39] He K., Zhang X., Ren S., Sun J. (2015). Spatial pyramid pooling in deep convolutional networks for visual recognition. IEEE Trans. Pattern Anal. Mach. Intel..

[40] Zheng X., Wang J., Shangguan L., Zhou Z., Liu Y. Smokey: Ubiquitous smoking detection with commercial wifi infrastructures. Proceedings of the IEEE INFOCOM 2016-The 35th Annual IEEE International Conference on Computer Communications.

[41] Qian K., Wu C., Zhou Z., Zheng Y., Yang Z., Liu Y. Inferring motion direction using commodity wi-fi for interactive exergames. Proceedings of the 2017 CHI Conference on Human Factors in Computing Systems.

[42] Rajwade A., Rangarajan A., Banerjee A. (2013). Image denoising using the higher order singular value decomposition. IEEE Trans. Pattern Anal. Mach. Intell..

[43] Brett W., Tamara G. MATLAB Tensor Toolbox Version 2.6. http://www.sandia.gov/~tgkolda/TensorToolbox/.

[44] Girshick R., Donahue J., Darrell T., Malik J. Rich feature hierarchies for accurate object detection and semantic segmentation. Proceedings of the 2014 IEEE Conference on Computer Vision and Pattern Recognition (CVPR).

[45] Uijlings J.R.R., Van De Sande K.E.A., Gevers T., Smeulders A. (2013). Selective search for object recognition. Int. J. Comput. Vis..

[46] Simonyan K., Andrew Z. (2014). Very deep convolutional networks for large-scale image recognition. arXiv preprint arXiv.

[47] Wang X., Yang C., Mao S. (2017). TensorBeat: Tensor Decomposition for Monitoring Multi-Person Breathing Beats with Commodity WiFi. ACM Trans. Intell. Syst. Technol..

[48] Szegedy C., Liu W., Jia Y., Sermanet P., Reed S., Anguelov D., Rabinovich A. Going deeper with convolutions. Proceedings of the 2015 IEEE Conference on Computer Vision and Pattern Recognition (CVPR).

[49] He K., Zhang X., Ren S., Sun J. Deep residual learning for image recognition. Proceedings of the 2016 IEEE Conference on Computer Vision and Pattern Recognition (CVPR).

